# Establishment of an Indirect ELISA Detection Method for Porcine Reproductive and Respiratory Syndrome Virus NSP10

**DOI:** 10.3390/vetsci13080793

**Published:** 2026-08-08

**Authors:** Gan Li, Qipeng Zhang, Xiaojing Chen, Huawei Li, Ruining Wang, Keshan Zhang, Yaqiong Ye, Mengmeng Zhao

**Affiliations:** 1 School of Animal Science and Technology, Foshan University, Foshan 528225, China; ligan1227@163.com (G.L.); zhangqp1236@163.com (Q.Z.); 20230420314@stu.fosu.edu.cn (X.C.); zks009@126.com (K.Z.); 2 Institute of Animal Product Quality and Safety Technology, Henan University of Animal Husbandry and Economy, Zhengzhou 450046, China; centrosome@126.com (H.L.); 80882@hnuahe.edu.cn (R.W.)

**Keywords:** PRRSV, *NSP10* gene, enzyme-linked immunosorbent assay, detection method

## Abstract

As a highly contagious infectious disease in the swine industry, porcine reproductive and respiratory syndrome (PRRS) can cause reproductive disorders in sows and respiratory diseases in piglets, resulting in sustained economic losses. Nonstructural protein 10 (NSP 10), as one of the core components of the replication and transcription complex of PRRS virus (PRRSV), has both helicase and adenosine triphosphatase activities, and has the potential as a diagnostic marker. In this study, NSP10 recombinant protein was used as coating antigen, and an indirect ELISA method for PRRSV NSP10 detection was established by square matrix titration. After optimizing the assay parameters, high specificity (no reactions with other pig viruses), high sensitivity (able to detect antibodies even when the positive blood sample was diluted 1280 times), and excellent reproducibility (less than 10% variation) were shown. Therefore, the NSP10 indirect enzyme-linked immunosorbent assay (ELISA) developed in this study provides an effective technical means for enhancing PRRSV surveillance in swine herds, thereby establishing a solid foundation for the clinical diagnosis and comprehensive prevention and control of PRRSV.

## Simple Summary

Nonstructural protein 10 (NSP10) is endowed with both helicase and adenosine triphosphatase activities and plays a crucial role in the replication and transcription of porcine reproductive and respiratory syndrome virus (PRRSV). In this study, NSP10 recombinant protein was used as the coating antigen to develop the NSP10 indirect enzyme-linked immunosorbent assay (ELISA) with high specificity, sensitivity and repeatability. This assay may facilitate early PRRSV diagnosis, contribute to outbreak prevention and control, and help reduce associated economic losses.

## 1. Introduction

Porcine reproductive and respiratory syndrome virus (PRRSV), the pathogen of porcine reproductive and respiratory syndrome (PRRS), causes reproductive failure in sows and respiratory disease in pigs of all ages [1]. Due to its rapid mutation rate, PRRSV can recombine with vaccine strains to generate new variants. Different strains vary in pathogenicity and produce distinct clinical symptoms upon infecting pigs. Moreover, PRRSV can participate in co-infections with other viruses [2,3,4]. Sick swine with suspected PRRS symptoms can only be diagnosed by laboratory testing. Virus isolation and identification, molecular biological detection and serological detection are the three main approaches for laboratory diagnosis of PRRSV [5]. Among them, virus isolation and identification is the clearest and most commonly used method to determine the virus species. The main method is to inoculate the lung tissue and positive serum with obvious pathological changes into porcine alveolar macrophage cells, CL2621 cells, ZMAC cells, HS2H cells or Marc-145 cells for passage separation [5].

Numerous molecular biological detection methods have been developed for virus identification. Polymerase chain reaction (PCR)-based techniques include real-time quantitative PCR (RT-qPCR), duplex PCR, one-step multiplex PCR, nested PCR, and reverse transcriptase PCR restriction fragment length polymorphism, all of which are applicable to various PRRSV sample types [5]. RT-qPCR can screen samples with low virus level, including ReTi RT-PCR, SYBR Green qPCR, Eva Green qPCR and TaqMan qPCR detection [6,7,8,9]. Loop-mediated isothermal amplification (LAMP) is widely used in pathogen detection due to its simple operation, low cost, and naked-eye visibility of results [10]. Xie et al. [11] established a double fluorescence LAMP detection method which can distinguish PRRSV from porcine circovirus type 2 (PCV2), and it has high specificity. In addition, some new detection methods that can improve the detection accuracy are emerging, such as digital PCR, recombinase polymerase amplification, clustered regularly interspaced short palindrome repeats, and virus metagenome sequencing [12,13,14,15,16].

The serological detection methods are based on tests using positive serum antibodies produced by pigs infected with PRRSV. This technique is relatively mature, and its basic principle is the specific binding of antigens and antibodies. It mainly includes indirect immunofluorescent assay (IFA), enzyme-linked immunosorbent assay (ELISA), and immunoperoxidase monolayer assay (IPMA) [17,18,19]. The commonly used method is ELISA, and there are many commercial kits, such as IDEXX in the United States, Jinnuo in South Korea, Keqian and Weideweikang kits in China, which can detect serum immunoglobulin G (IgG) antibodies [20]. Nonstructural protein 10 (NSP10), as the core enzyme component of the viral replication and transcription complex [21,22], exhibits a high degree of sequence conservation between PRRSV-1 and PRRSV-2 and contains multiple B-cell and T-cell epitopes, making it an ideal target for the development of diagnostic reagents and antiviral drugs. In this study, the *NSP10* gene fragment was amplified from the PRRSV NADC30 strain using PCR and ligated into the pET28a (+) vector to successfully construct the recombinant plasmid pET28a-NSP10. The recombinant protein was purified, renatured, and concentrated after verification by sodium dodecyl sulfate–polyacrylamide gel electrophoresis (SDS-PAGE) and Western blotting. The reaction conditions were optimized, and the specificity, sensitivity, and repeatability of the assay were evaluated and compared with those of the PRRSV IDEXX ELISA kit. An indirect ELISA detection method based on PRRSV NSP10 was thereby established, and it exhibits high specificity, sensitivity, and repeatability, which can effectively reduce the detection cost of PRRSV.

## 2. Materials and Methods

### 2.1. Construction of Recombinant Expression Plasmid

Based on the sequence of the NADC30 *NSP10* gene, a pair of expression primers were designed (F: 5′-CGCGGATCCGGGAAGAAGTCAAGGGTGTG-3′, R: 5′-CCGCTCGAGATTCCAGATCTGCACATATGGC-3′). After pre-denaturation at 98 °C for 30 s, amplification was performed for 35 cycles (98 °C for 10 s, 56 °C for 30 s, 72 °C for 2 min), followed by a final extension at 72 °C for 10 min, yielding a 1323 bp amplicon. The pET28a (+) vector was selected for recombinant protein expression, with the restriction enzymes *Bam*H I (GGATCC) (Takara Bio Inc., Dalian, China) and *Xho* I (CTCGAG) (Takara Bio Inc., Dalian, China) chosen for cloning. The purified PCR products and the pET28a (+) vector were separately digested at 37 °C for 6 h. Following recovery and purification, the digested fragments were ligated using T4 DNA ligase (New England Biolabs Ltd., Beijing, China) at 4 °C overnight. The ligation product was transformed into *Escherichia coli* (*E. coli*) *DH5α* under aseptic conditions, and cultured at 37 °C for 12 h, then a single colony was selected and inoculated in Luria–Bertani agar plates containing Kanamycin (50 mg·L^−1^) resistance for culture, and the bacterial liquid identified as positive was subjected to plasmid extraction, double enzyme digestion identification and sequencing.

### 2.2. Target Gene Expression, Purification, and Validation

The sequence-verified plasmid was transformed into *BL21(DE3)* competent cells. A single colony was selected for culture, and isopropyl-*β*-D-galactoside (IPTG) was added to induce expression. Different induction times, concentrations and temperatures were set to explore the best induction conditions. After induction, the bacterial pellet was collected by centrifugation at 4 °C and 4000 rpm for 10 min, resuspended in pre-chilled phosphate-buffered saline, and then disrupted by sonication. The pellet and supernatant were separately collected for analysis, and based on the results, an appropriate purification method was selected to obtain recombinant protein with high purity.

### 2.3. Optimization of Indirect ELISA Conditions and Cut-Off Value Determination

The NSP10 recombinant protein (0.25, 0.5, 1.0, 2.0, 4.0, and 8.0 mg·L^−1^) was diluted in 1× coating solution, used to coat the microtiter plates, and incubated overnight at 4 °C. The plates were then washed 5 times with phosphate-buffered saline with Tween 20. The negative and positive PRRSV serum used as primary antibody were diluted 1:50 to 1:1600, respectively, and the optimal antigen coating concentration and serum dilution multiple were determined. Subsequently, blocking solutions containing bovine serum albumin, fetal bovine serum, and skimmed milk were prepared at three concentration gradients of 1.0%, 2.0%, and 3.0%. Three blocking times (1, 2, and 3 h) were tested at 37 °C to determine the optimal blocking solution and blocking time. Five primary antibody incubation times (30, 45, 60, 75, and 90 min), four dilutions of horseradish peroxidase (HRP)-conjugated goat anti-pig IgG (1:5000, 1:10,000, 1:15,000, and 1:20,000), and four secondary antibody incubation times (30, 60, 90, and 120 min) were set to determine the optimal primary antibody incubation time, secondary antibody dilution, and incubation time. Meanwhile, based on the optimal conditions established above, the optimal chromogenic time (5, 10, 15, and 20 min) was determined. Finally, the cut-off value for distinguishing positive and negative results was determined according to the method of Zhao et al. [23].

### 2.4. Evaluation of Assay Specificity, Sensitivity and Repeatability

The specificity of the assay was evaluated by testing positive sera against PCV2, PRRSV, pseudorabies virus (PRV), Japanese encephalitis virus (JEV), African swine fever virus (ASFV), and classical swine fever virus (CSFV), as well as other common swine pathogens. The PRRSV standard positive serum was diluted (1:10 to 1:5120) to evaluate the sensitivity. The same batch and different batches of purified and renatured NSP10 recombinant protein were used to coat the enzyme-labeled plate, and PRRSV serum samples (4 positive sera and 4 negative sera) were randomly selected for the test, and the coefficient of variation was calculated to evaluate the repeatability within and between batches.

### 2.5. Comparative Analysis of the Indirect ELISA with the IDEXX ELISA Kit

A total of 230 porcine serum samples (70 from Jiangmen, 58 from Zhanjiang, 47 from Qingyuan, and 55 from Shaoguan) were collected from swine suspected of PRRSV infection. All samples were identified by IDEXX ELISA kit and NSP10 indirect ELISA developed in this study. The results were analyzed, and the concordance rate between the two assays was calculated. Receiver operating characteristic (ROC) curves were plotted using GraphPad Prism 9.0 software, and Cohen’s kappa coefficient was calculated via the GraphPad online calculator (https://www.graphpad.com/quickcalcs/kappa1/) (accessed on 24 July 2026) [24], to comprehensively evaluate the diagnostic performance of the NSP10 indirect ELISA.

## 3. Results

### 3.1. Construction and Identification of Recombinant Plasmid pET28a-NSP10

**Figure 1:**
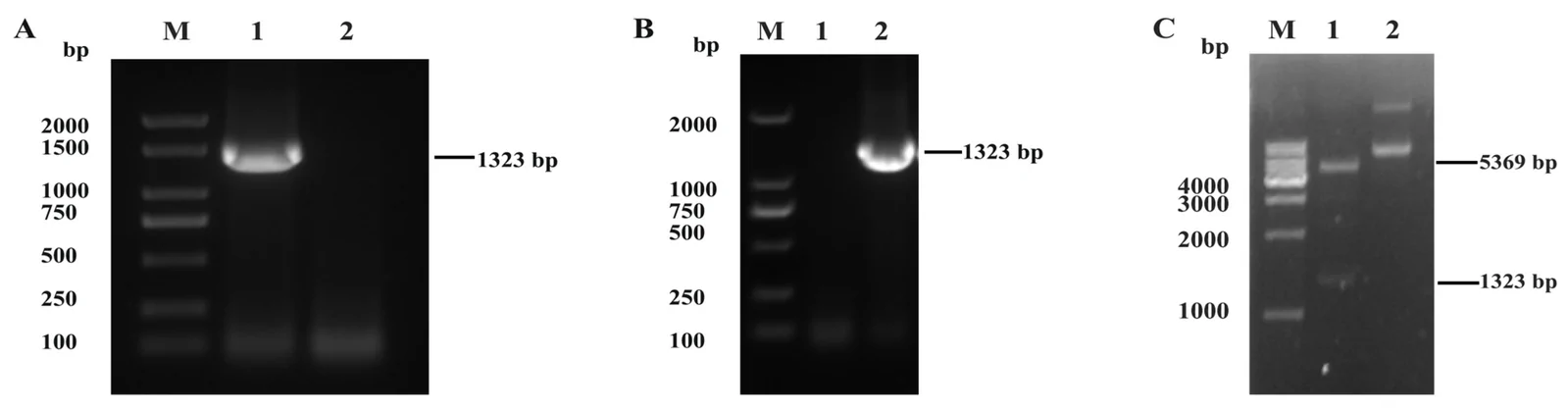
Construction and identification of recombinant plasmid pET28a-NSP10. (**A**) Amplification results of NSP10 gene fragment of NADC30 strain. M: D2000 DNA Marker; 1: PCR amplification products; 2: negative control. (**B**) PCR amplification results of PET28a-NSP10 bacterial solution. M: D2000 DNA Marker; 1: negative control; 2: pET28a-NSP10 bacterial solution. (**C**) Identification results of recombinant plasmid by double enzyme digestion. M: D10,000 DNA Marker; 1: double enzyme digestion products; 2: recombinant plasmid pET28a-NSP10 (the original Western-blot pictures can be found in File S1).

The *NSP10* gene was amplified by using NADC30 cDNA as a template (Figure 1A), and then it was connected to pET28a (+) vector, and then it was identified by bacterial liquid PCR (Figure 1B) and double restriction enzyme digestion (Figure 1C). The sequence consistent with the expected insert was obtained by sequencing, which further proved the successful construction of the recombinant plasmid pET28a-NSP10.

### 3.2. Expression and Verification of NSP10 Recombinant Protein

**Figure 2:**
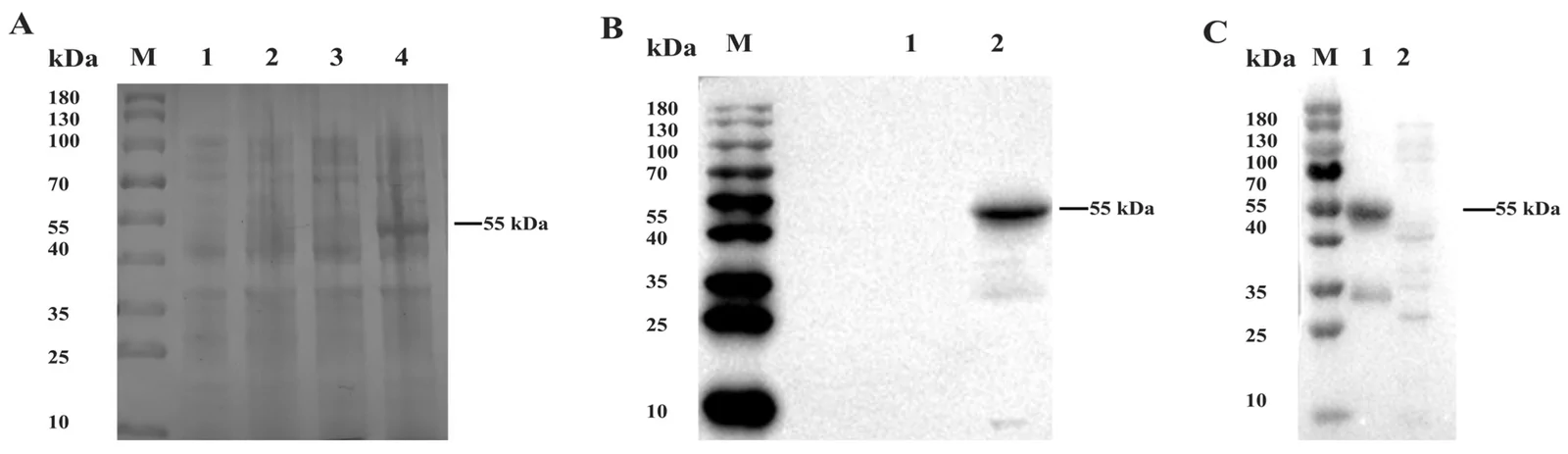
Identification of NSP10 recombinant protein expression. (**A**) Identification of NSP10 recombinant protein by SDS-PAGE electrophoresis. M: 180 kDa protein Marker; 1: pET28a (+) empty vector (not induced); 2: pET28a (+) empty carrier (induced); 3: pET28a-NSP10 (not induced); 4: pET28a-NSP10 (induced). (**B**) Western blotting verified the expression result of NSP10 protein (primary antibody: His-tag (10E2) mAb). M: 180 kDa protein Marker; 1: pET28a (+) empty vector; 2: pET28a-NSP10. (**C**) Western blotting verified the expression result of NSP10 protein (primary antibody: PRRSV positive serum). M: 180 kDa protein Marker; 1: pET28a-NSP10; 2: pET28a (+) empty vector (the original Western-blot pictures can be found in File S1).

The NSP10 recombinant protein was transformed into *BL21(DE3)* competent cells, and after 7 h of IPTG induction, SDS-PAGE electrophoresis analysis showed that there was a protein band at about 55 kDa (Figure 2A). Solubility analysis showed that NSP10 recombinant protein existed in the form of inclusion bodies. After purification and renaturation under denaturing conditions, His-tag (10E2) mAb and PRRSV positive serum were used as primary antibodies (Figure 2B,C). Western blotting showed that NSP10 recombinant protein could be specifically recognized by these two antibodies and had strong immunoreactivity.

### 3.3. Optimization of Indirect ELISA Reaction Conditions for PRRSV NSP10

**Figure 3:**
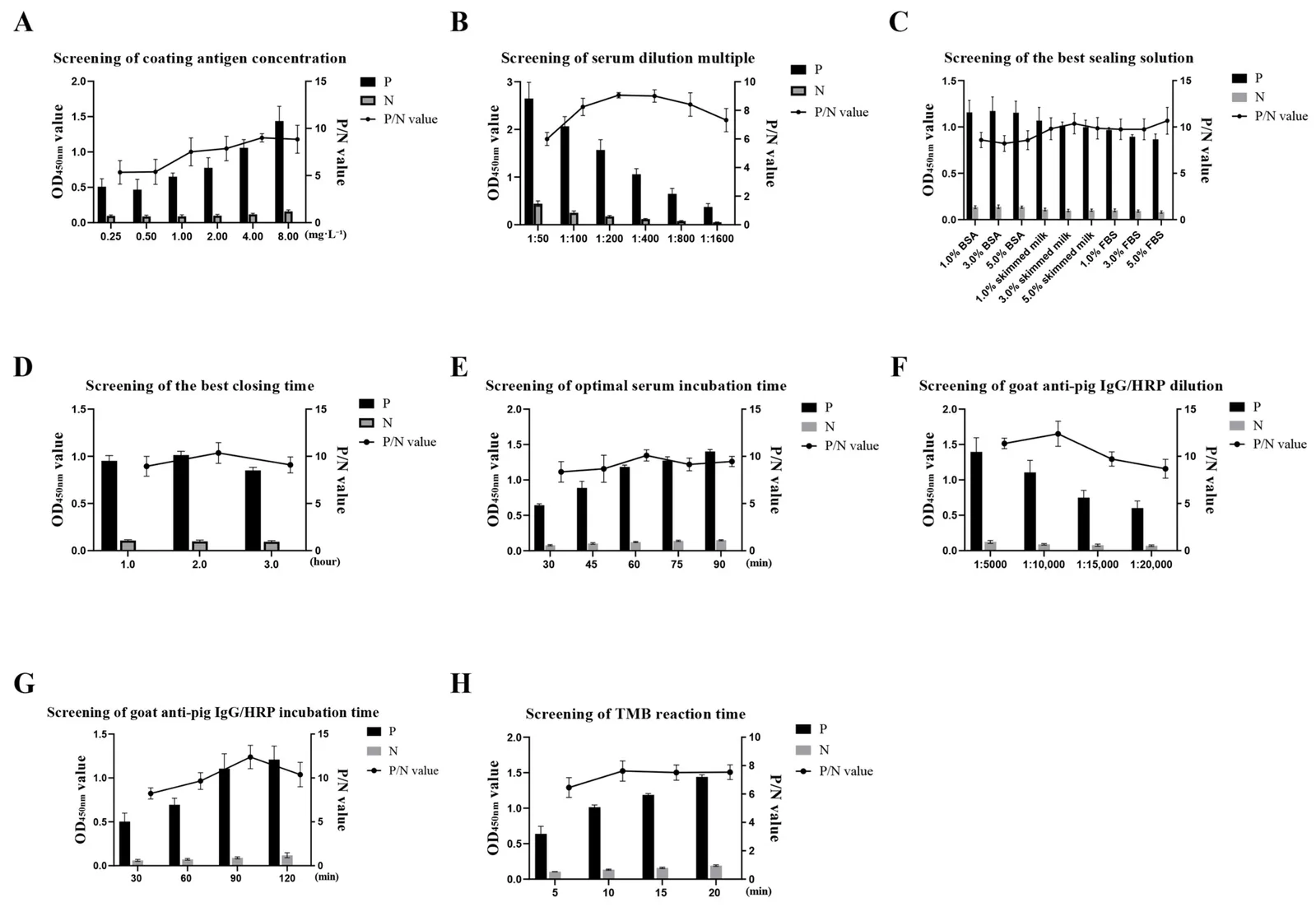
Optimization of reaction conditions for the PRRSV NSP10 indirect ELISA detection method. (**A**) Optimal antigen coating concentration. (**B**) Serum dilution multiple. (**C**) Optimal blocking solution. (**D**) Optimal blocking time. (**E**) Optimal serum incubation time. (**F**) Goat anti-pig IgG/HRP dilution. (**G**) Goat anti-pig IgG/HRP incubation time. (**H**) Tetramethylbenzidine (TMB) reaction time.

An indirect ELISA method was successfully established using the recombinant NSP10 protein as the coating antigen. The reaction parameters were optimized, and the optimal reaction conditions were determined by the criterion that the *p* value approached 1 and the P/N value was the maximum. The optimal signal-to-noise ratio was obtained under the following conditions: the plate was coated with antigen at 4.0 mg·L^−1^, blocked with 3.0% skimmed milk for 2 h, and incubated sequentially with serum diluted 1:400 for 1 h at 37 °C and with goat anti-pig IgG/HRP diluted 1:10,000 for 90 min, followed by color development for 10 min (Figure 3). Based on the above optimum reaction conditions, 40 PRRSV negative serum samples were detected and the results were statistically analyzed. The results showed that the average value (X) of 40 serum samples at OD_450nm_ was 0.177, and the standard deviation (SD) was 0.055, from which the positive critical value ($\bar{x}$ + 3SD) was 0.358 and the negative critical value ($\bar{x}$ + 2SD) was 0.299. Samples with OD_450nm_ values between 0.299 and 0.358 were considered equivocal and subjected to retesting.

### 3.4. Evaluation of Specificity, Sensitivity and Repeatability of Indirect ELISA

**Figure 4:**
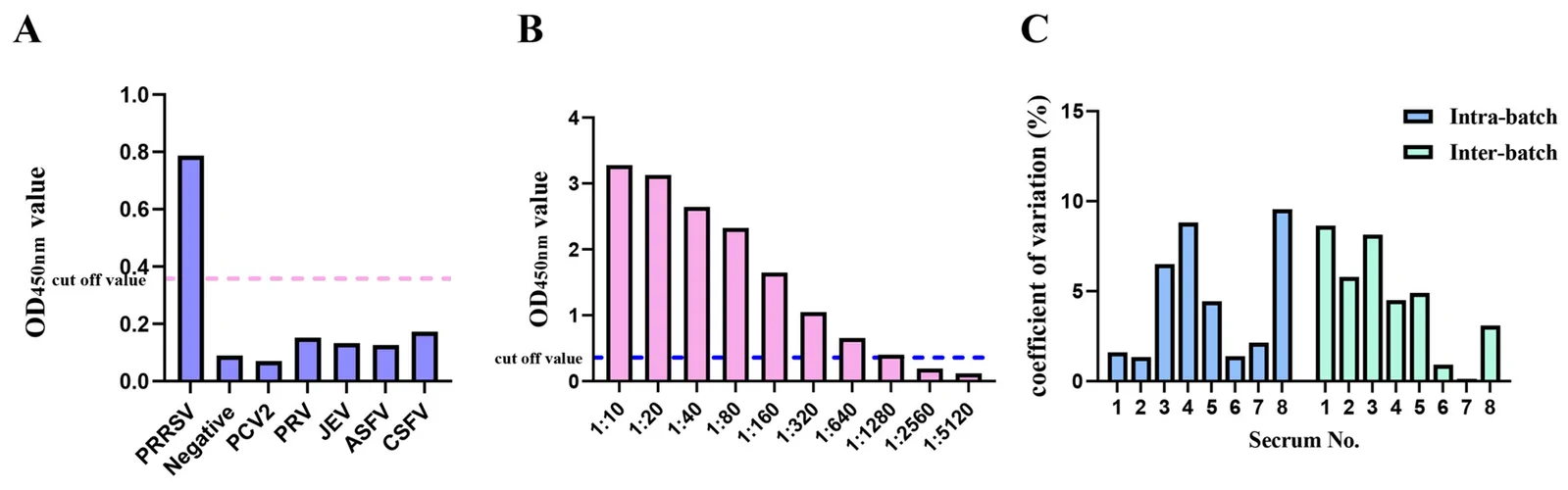
Specificity, sensitivity and repeatability of indirect ELISA. (**A**) Specificity of indirect ELISA detection method. (**B**) Sensitivity of indirect ELISA detection method. (**C**) Repeatability of indirect ELISA detection method.

According to the established NSP10 indirect ELISA detection method, a variety of swine pathogen positive sera (PCV2, PRV, JEV, ASFV, CSFV) were detected. The results showed that only the OD_450nm_ value of PRRSV positive serum was higher than 0.358, while the OD_450nm_ values of other virus positive sera and PRRSV negative sera were lower than 0.299, which indicated that the indirect ELISA detection method had high specificity and had no cross reaction with other pathogen positive sera (Figure 4). When the PRRSV-positive serum was diluted 1280-fold, the result remained positive, indicating that the method is highly sensitive and capable of detecting the PRRSV NSP10 antibody even at such a high dilution. In addition, the results of intra-batch and inter-batch repeatability tests showed that the coefficient of variation is less than 10%, indicating that the detection method has high repeatability.

### 3.5. Comparison of Conformity Rate with IDEXX ELISA Kit

**Figure 5:**
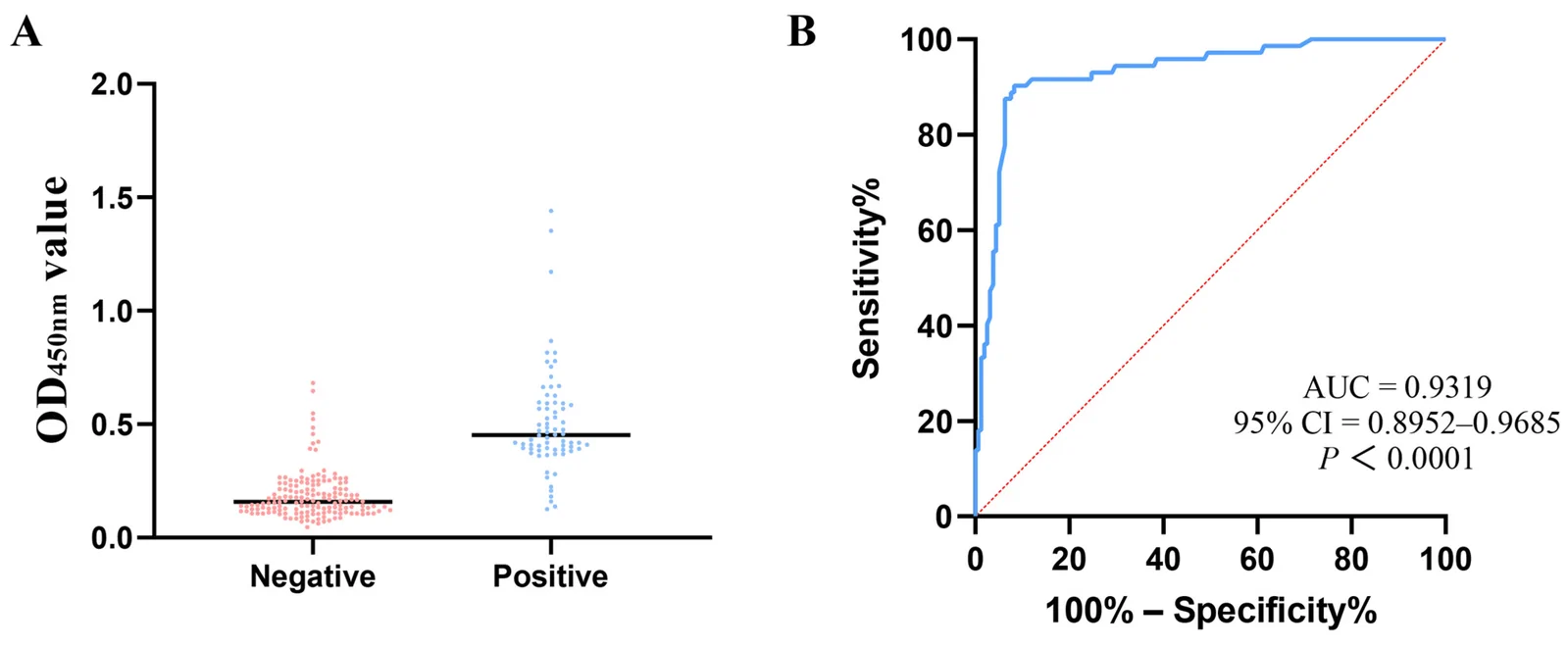
Receiver operating characteristic (ROC) analysis for the NSP10 indirect ELISA. (**A**) Distribution of OD_450nm_ values of porcine serum samples detected by the NSP10 indirect ELISA. (**B**) ROC curve of the NSP10 indirect ELISA, AUC = 0.9319.

A total of 230 swine serum samples were evaluated using the developed indirect NSP10 ELISA (Figure 5A) and a commercially available PRRSV IDEXX ELISA kit. The results showed that 73 positive and 157 negative samples were identified by the developed indirect NSP10 ELISA (Table 1), while the PRRSV IDEXX ELISA kit flagged 72 positive samples and 158 negative samples. Among them, 63 samples were positive and 148 were negative by both methods. It is calculated that the positive concordance rate of this method with IDEXX ELISA kit is 87.5%, the negative concordance rate is 93.7%, and the total concordance rate is 91.7%.

The diagnostic accuracy of the NSP10 indirect ELISA was evaluated using ROC curve analysis and Cohen’s kappa coefficient. The ROC curve analysis yielded an AUC of 0.9319 with a 95% confidence interval (CI) of 0.8952–0.9685 (Figure 5B), a sensitivity of 87.5% (95% CI: 0.7792–0.9328), a specificity of 93.67% (95% CI: 0.8874–0.9653), and a kappa value of 0.809 (95% CI: 0.726–0.891). Collectively, these findings indicate excellent diagnostic performance of the NSP10 indirect ELISA.

## 4. Discussion

Since PRRSV was first identified in the United States in 1987, it has been detected in numerous countries, causing substantial economic losses to the global swine industry [25]. Consequently, rapid and effective detection methods are critical for monitoring and preventing the spread of PRRSV [26]. Collins et al. and Chen et al. [27,28] successfully detected PRRSV by the IFA method, which provided a powerful tool for clinical diagnosis. IPMA is applied to PRRSV antibody detection because of its simple operation, low equipment requirements and no need for a large number of purified antigens [29]. With the deepening of the research on PRRSV, molecular biological detection technologies have evolved from RT-PCR, RT-qPCR and LAMP to digital PCR, nano-PCR, recombinase polymerase amplification, clustered regularly interspaced short palindrome repeats, and metagenomic high-throughput sequencing [30], which can effectively realize the accurate typing and effective monitoring of PRRSV. After swine are infected with PRRSV, the body produces antibodies against different viral proteins. ELISA, based on antigen–antibody binding reactions, enables rapid, efficient, and large-scale detection, offering advantages such as high specificity, high sensitivity, and simple operation [31]. Currently, the commonly used ELISA detection kits for PRRSV mainly target the glycoprotein 5 and nucleocapsid proteins. In addition, indirect ELISA methods have also been developed using non-structural proteins such as NSP4, NSP7, and NSP12 as coating antigens [23,32,33], all of which exhibit high specificity, sensitivity, and repeatability. The development of these kits demonstrates that proteins appearing only during viral replication can also be used to construct relevant detection methods and effectively identify PRRSV-positive sera. Multiple antigenic epitopes and strong immunogenicity are associated with the NSP10 protein [34,35], rendering it a potential target for the development of serological detection tools. In contrast to other NSPs, NSP10 possesses both helicase and adenosine triphosphatase activities, which enable it to unwind double-stranded RNAs and drive viral genome replication, subgenomic mRNA transcription, and subgenomic RNA synthesis [36,37,38]. Hence, NSP10 is a critical early-stage regulator in the viral replication cycle.

At present, there are few detection methods targeting the PRRSV *NSP10* gene. In this study, the recombinant plasmid pET28a-NSP10 was successfully constructed using the expression vector pET28a (+) and was transformed into the prokaryotic expression *E. coli BL21 (DE3)*. After induction of expression and optimization of conditions, it was determined that the recombinant protein exhibited the highest expression level under conditions of 37 °C with 0.8 mmol·L^−1^ IPTG induction for 7 h, and was then subjected to SDS-PAGE and examined. Therefore, after dissolving the recombinant protein in a denaturing eluent containing 8 M urea, a stepwise renaturation was performed using renaturation buffers containing decreasing concentrations of urea (6.0 M, 4.0 M, 2.0 M, 1.0 M, and 0 M) to promote the refolding of the denatured inclusion body protein under mild conditions, thereby restoring its native conformation and biological activity. The protein was then concentrated at 4 °C, ultimately yielding a high-purity product that appeared as a single band at approximately 55 kDa on SDS-PAGE (Figure 2B,C).

Based on this, the NSP10 recombinant protein was used as an antigen to construct indirect ELISA detection method, and the optimum reaction conditions were determined (Figure 3). Different virus-positive sera were used for specificity determination, and the concordance rate was compared with 230 serum samples detected by commercial IDEXX ELISA kit. The results showed that an indirect ELISA method for NSP10 detection with high sensitivity, specificity and repeatability was successfully established (Figure 4), which had a high concordance rate with the detection results of the IDEXX ELISA kit (Table 1). Furthermore, according to the criteria of Mandrekar et al. [39], an AUC > 0.9 indicates high diagnostic accuracy. The NSP10 indirect ELISA had an AUC of 0.9319 and a kappa value of 0.809 (Figure 5B), confirming the strong diagnostic performance of this assay. As a helicase, the NSP10 protein is mainly involved in the process of virus replication and synthesis. Regular detection of NSP10 antibody level can monitor PRRSV infection over time. The NSP10 protein has been demonstrated to be highly conserved in previous studies [40], with relatively few amino acid mutation sites and high sequence similarity being observed among different strains. Therefore, it is speculated that the PRRSV NSP10 indirect ELISA method established may be applicable to the diagnosis of infections caused by multiple strains. Further validation and evaluation are expected to be conducted in subsequent studies to test this hypothesis.

Further optimization of the indirect ELISA method for NSP10 is required, particularly regarding the fresh dilution of the goat anti-pig IgG/HRP immediately before use. To simplify the procedure and enhance convenience, efforts should be made to determine how to dilute the secondary antibody to its working concentration during the production stage. In addition, variability in the detection results was observed following repeated freeze–thaw cycles of serum samples. It was speculated to be attributable to reduced antigen-binding capacity of serum antibodies or protein aggregation that promoted nonspecific binding, which would ultimately compromise the accuracy and reproducibility of the assay. Therefore, serum samples should be aliquoted before use to minimize these confounding factors. Based on high specificity, sensitivity and repeatability, the NSP10 indirect ELISA constructed in this study has the advantages of low cost and simple operation, and is suitable for large-scale PRRSV detection, which can provide strong support for early PRRSV diagnosis, better prevent and control PRRSV outbreaks and reduce economic losses.

## 5. Conclusions

In this study, the PRRSV NSP10 recombinant protein was successfully expressed, and it was used as a coating antigen to establish an indirect ELISA characterized by high specificity, sensitivity, and reproducibility. The total concordance rate between this method and the IDEXX ELISA kit was determined to be 91.7%. Moreover, the assay was characterized by operational simplicity and cost-effectiveness, thereby exhibiting considerable potential for widespread application. In conclusion, the indirect ELISA for PRRSV NSP10 established in this study is expected to reduce the economic costs of PRRSV detection. It is considered to be of substantial veterinary public health significance, as it contributes to the enhanced precision prevention and control of PRRS in Chinese swine herds, thereby supporting the healthy and sustainable development of the pig industry.

## Figures and Tables

**Table 1:** The concordance test results between the developed indirect NSP10 ELISA and the commercial kit.

Detection Method	IDEXX ELISA Commercial Kit		
	Positive (*n*)	Negative (*n*)	Total (*n*)
Positive samples tested by the NSP10 indirect ELISA	63	10	73
Negative samples tested by the NSP10 indirect ELISA	9	148	157
Total (*n*)	72	158	230
Concordance rate	87.5%	93.7%	91.7%

## Data Availability

The original contributions presented in this study are included in the article/Supplementary Material. Further inquiries can be directed to the corresponding authors.

## Supplementary Materials

The following supporting information can be downloaded at: https://www.mdpi.com/article/10.3390/vetsci13080793/s1, File S1: Original images of SDS-PAGE and Western-blotting.

## Abbreviations

The following abbreviations are used in this manuscript:

PRRS	Porcine reproductive and respiratory syndrome
PRRSV	Porcine reproductive and respiratory syndrome virus
NSP10	Nonstructural protein 10
PCR	Polymerase chain reaction
RT-qPCR	Real-time quantitative PCR
IFA	Immunofluorescent assay
ELISA	Enzyme-linked immunosorbent assay
IgG	Immunoglobulin G
IPMA	Immunoperoxidase monolayer assay
SDS-PAGE	Sodium dodecyl sulfate–polyacrylamide gel electrophoresis
LAMP	Loop-mediated isothermal amplification
*E. coli*	*Escherichia coli*
IPTG	Isopropyl-β-D-galactoside
HRP	Horseradish peroxidase
PCV2	Porcine circovirus type 2
PRV	Pseudorabies virus
JEV	Japanese encephalitis virus
ASFV	African swine fever virus
CSFV	Classical swine fever virus
ROC	Receiver operating characteristic
SD	Standard deviation
CI	Confidence interval
