# Supplementary material for: Establishment of an Indirect ELISA Detection Method for Porcine Reproductive and Respiratory Syndrome Virus NSP10
Source: Vet Sci. 2026 Aug 8;13(8):793. doi: 10.3390/vetsci13080793 (PMC13517849; doi:10.3390/vetsci13080793)
Supplement: Supplementary file 1 [file vetsci-13-00793-s001.zip › Supplementary Materials.pdf]

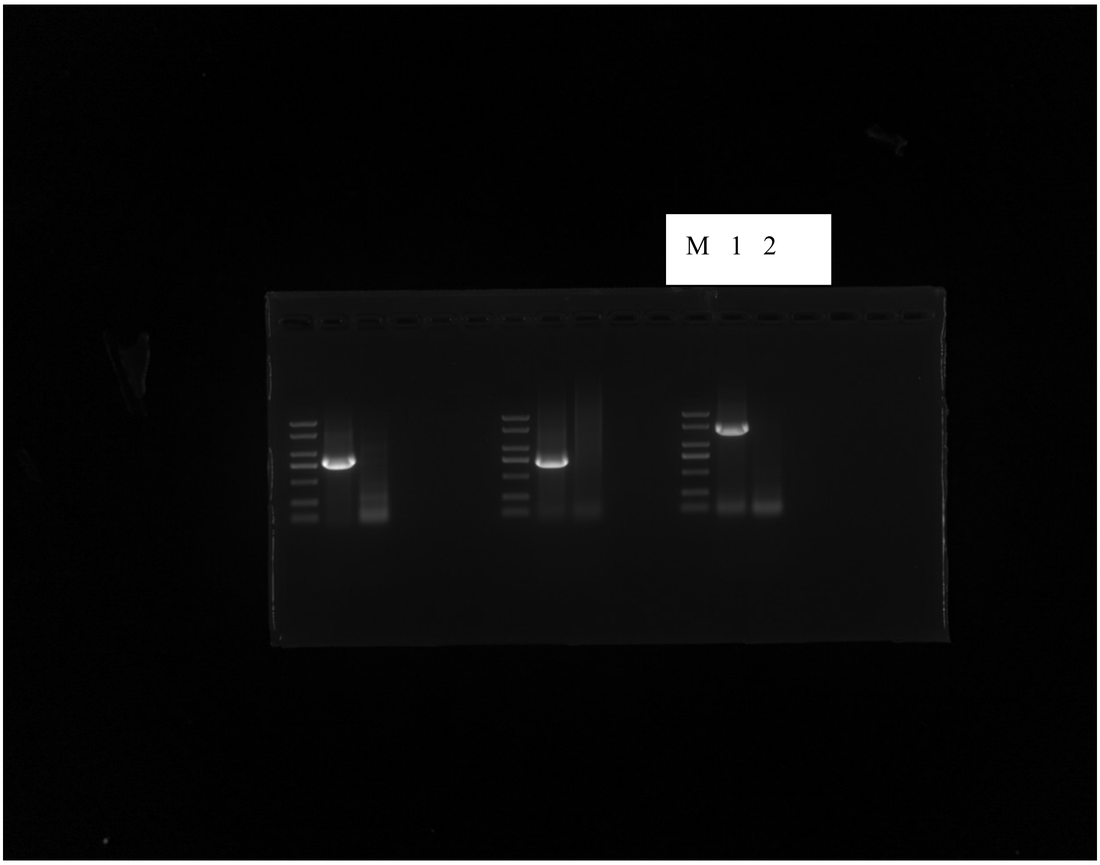

Figure S1A. Amplification results of *NSP10* gene fragment of NADC30 strain. M: D2000 DNA Marker; 1: PCR amplification products; 2: Negative control.

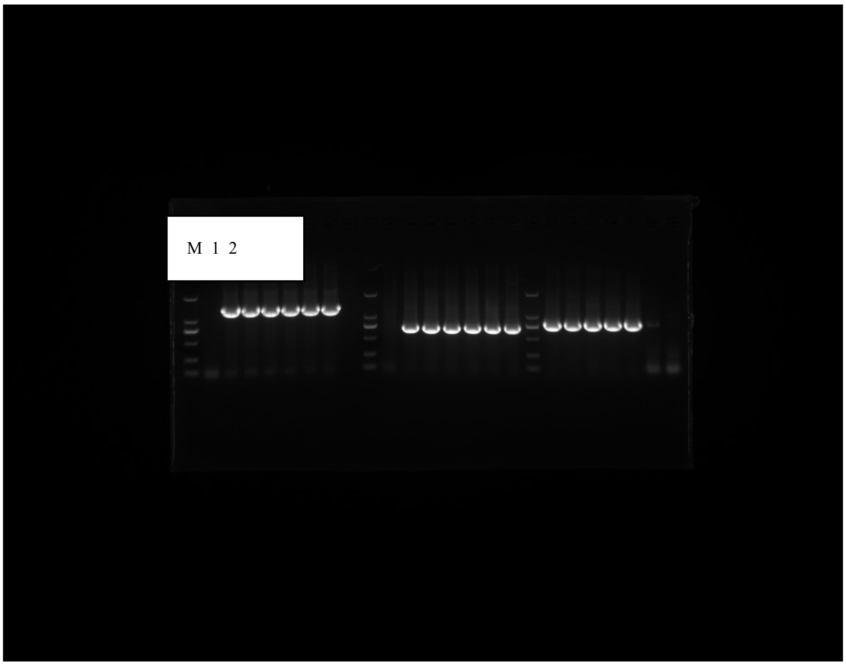

Figure S1B. PCR amplification results of PET28a-NSP10 bacterial solution. M: D2000 DNA Marker; 1: negative control; 2: pET28a-NSP10 bacterial solution.

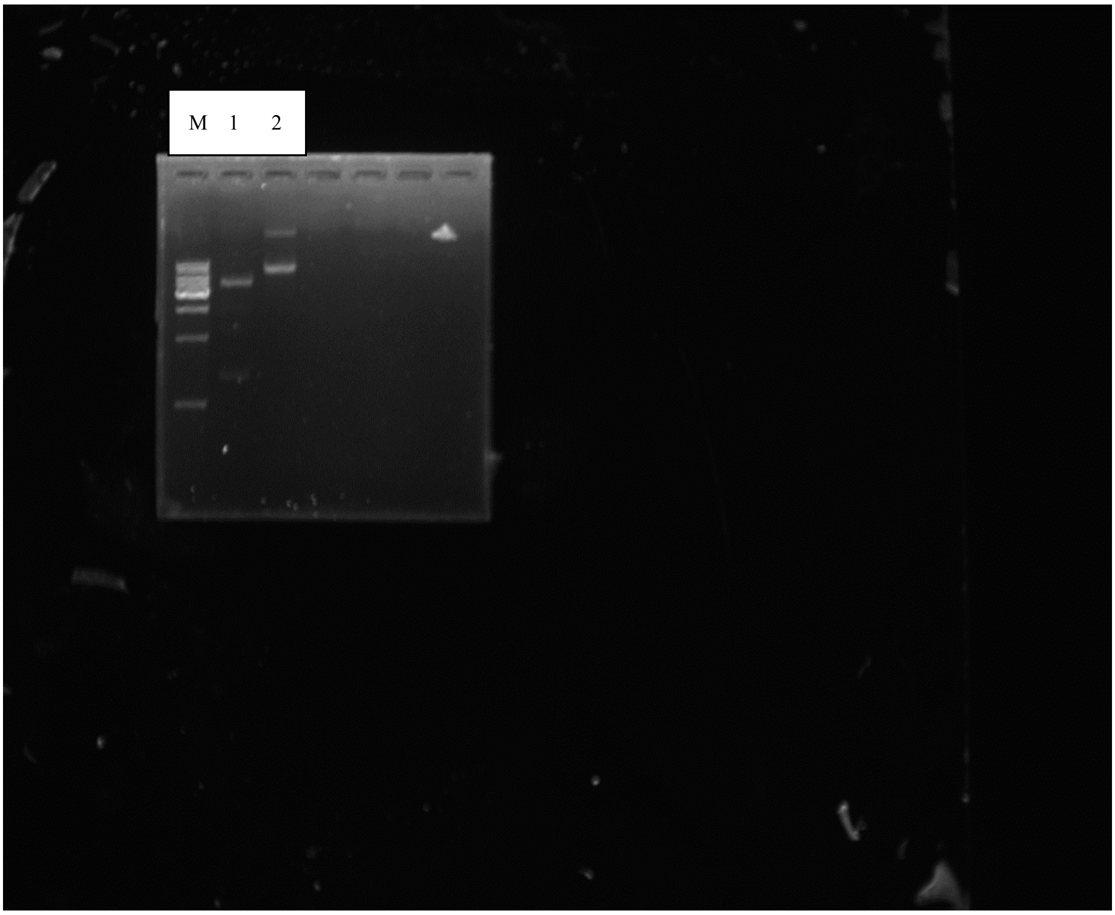

Figure S1C. Identification results of recombinant plasmid by double enzyme digestion. M: D10000 DNA Marker; 1: Double enzyme digestion products; 2: recombinant plasmid pET28a-NSP10.

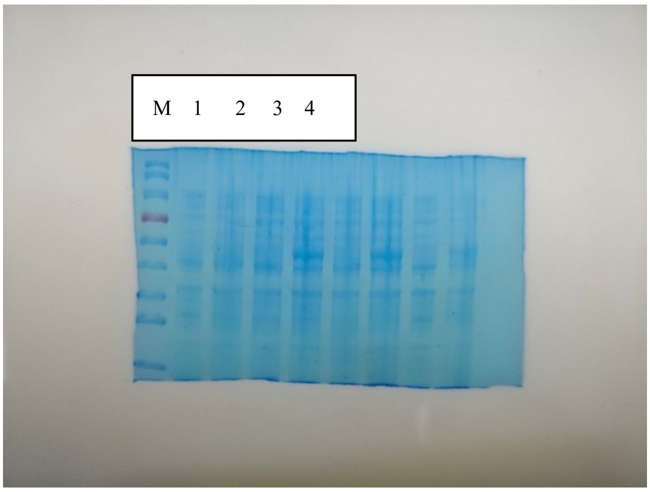

Figure S2A. Identification of NSP10 recombinant protein by SDS-PAGE electrophoresis. M: 180 kDa protein Marker; 1: pET28a (+) empty vector (not induced); 2: PET28a (+) empty carrier (induced); 3: pET28a-NSP10 (not induced); 4: pET28a-NSP10 (induced).

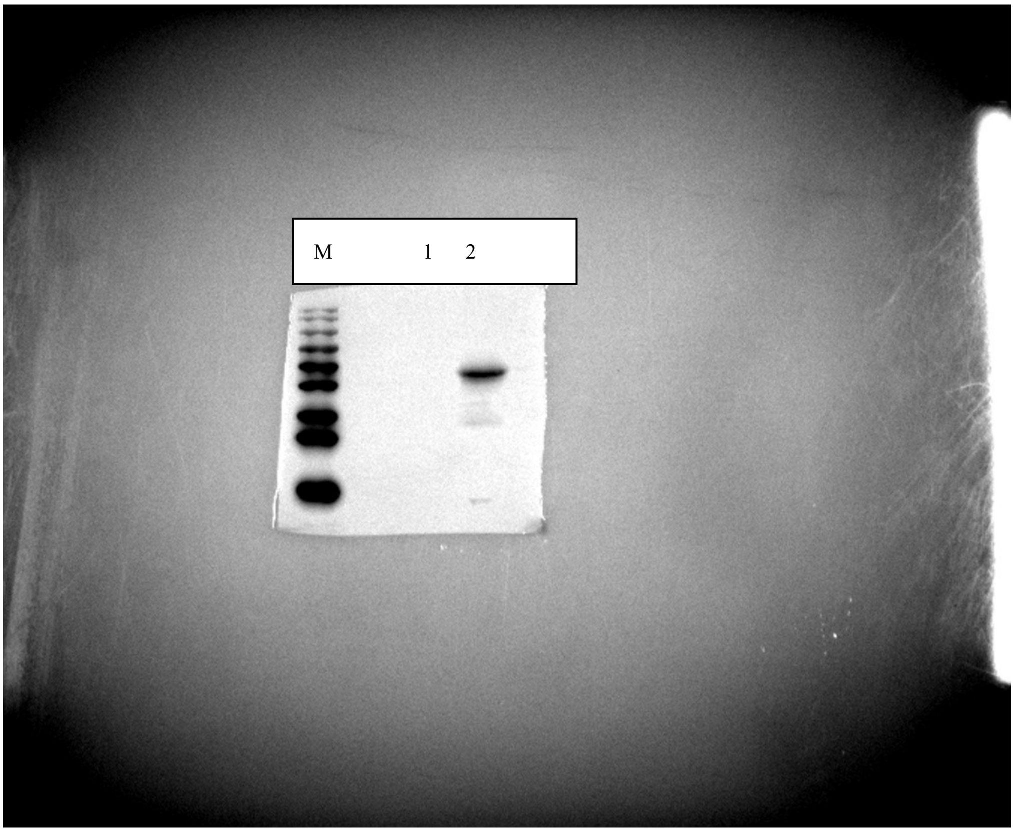

Figure S2B. Western blotting verified the expression result of NSP10 protein (primary antibody: His-tag (10E2) mAb). M: 180 kDa protein Marker; 1: pET28a (+) empty vector; 2: pET28a-NSP10.

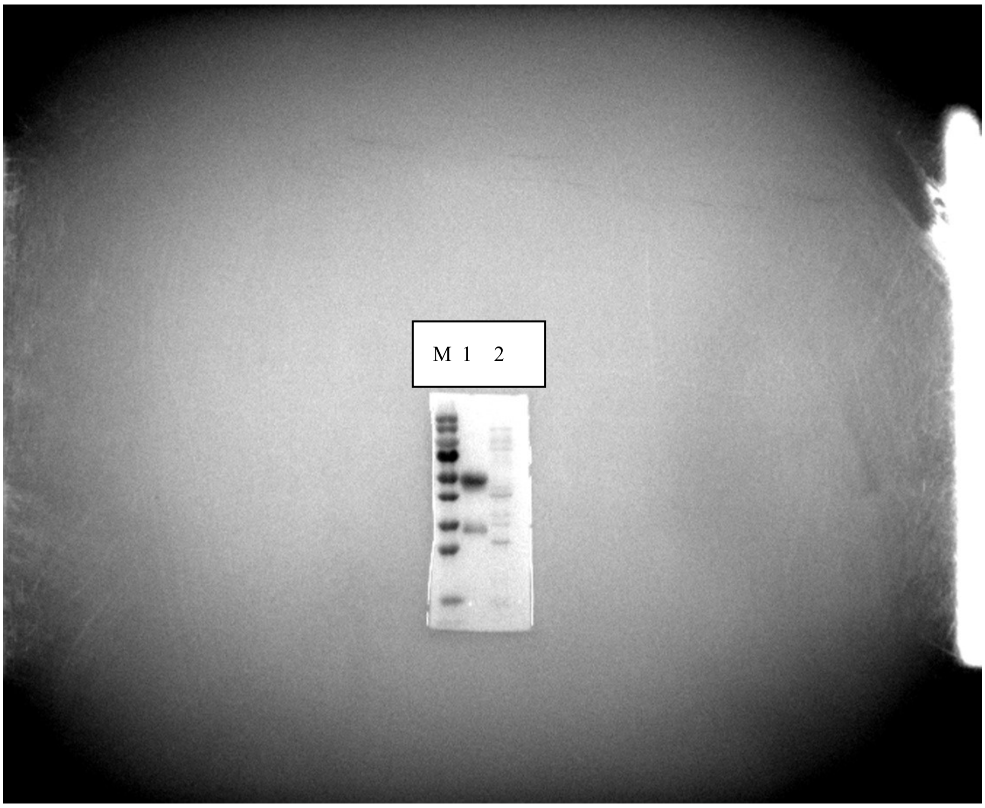

Figure S2C. Western blotting verified the expression result of NSP10 protein (primary antibody: PRRSV positive serum). M: 180 kDa protein Marker; 1: pET28a-NSP10; 2: pET28a (+) empty vector.
